# Glycemic Control and Prostate Cancer Mortality Risk in Veterans with Type 2 Diabetes Mellitus

**DOI:** 10.1158/2767-9764.CRC-25-0037

**Published:** 2025-08-01

**Authors:** Kinfe G. Bishu, Andrew D. Schreiner, Nicholas Shungu, Vanessa A. Diaz, Macelyn Batten, Mulugeta Gebregziabher

**Affiliations:** 1 Ralph H. Johnson VA Medical Center, Charleston, South Carolina.; 2 Department of Internal Medicine, Medical University of South Carolina, Charleston, South Carolina.; 3 Department of Family Medicine, Medical University of South Carolina, Charleston, South Carolina.; 4 Department of Public Health Sciences, Medical University of South Carolina, Charleston, South Carolina.

## Abstract

This retrospective cohort study of veterans diagnosed with diabetes mellitus evaluated the association between time-varying measures of glycemic control and the time to prostate cancer–specific mortality. Competing risk Cox regression models were developed to estimate the association of glycemic control and prostate cancer mortality for the entire sample and stratified by racial and ethnic groups. A total of 763,424 veterans with type 2 diabetes mellitus (T2DM) were included. In the fully adjusted models, moderate glycemic control [hemoglobin A1c (HbA1c) 7%–8%] was associated with a 23% (HR, 0.77; 95% confidence interval, 0.68–0.85) lower risk and poor glycemic control (HbA1c >8%) was associated with a 15% (HR, 0.85; 95% confidence interval, 0.71–0.99) lower risk of prostate cancer mortality compared with good glycemic control (HbA1c <7%), respectively. In the analyses stratified by race and ethnicity, moderate glycemic control was associated with a lower risk of prostate cancer mortality in non-Hispanic White and non-Hispanic Black veterans.

**Significance:**

Unlike many other cancers, there is an inverse association between prostate cancer risk and T2DM diagnosis. In this large, retrospective study of male veterans with T2DM, we observed an inverse association between glycemic control and prostate cancer mortality. Further research is required to verify this relationship in prospective studies and identify the potential mechanisms contributing to these findings.

## Introduction

The prevalence of diagnosed type 2 diabetes mellitus (T2DM) in the United States has increased over the last two decades and now affects an estimated 14.7% of all adults and 22% of all veterans (1, 2). In addition to its well-known relationships with cardiovascular, liver, and kidney diseases, T2DM is also associated with an increased risk of many cancers (3–5). Hyperinsulinemia, hyperglycemia, and inflammation provide the mechanistic link between T2DM and cancer occurrence, whereas risk factors shared by T2DM and cancer (e.g., obesity and dietary exposures such as processed foods and refined sugars) also likely contribute to this relationship (6, 7). Specifically, T2DM is associated with an increased risk of colorectal, pancreatic, hepatocellular, hepatobiliary, ovarian, and endometrial cancers (8). Knowledge of this heightened cancer risk has led to stronger guidance on routine cancer screening for patients receiving diabetes care (9).

Curiously, the relationship between T2DM and prostate cancer does not adhere to the associations observed with other site-specific cancers (10). Multiple studies have demonstrated that compared with men without T2DM, those with diabetes have a lower incidence of prostate cancer (11, 12). However, it is oversimplistic to suggest that T2DM protects patients from prostate cancer, because men with T2DM who do go on to develop prostate cancer have a higher prostate cancer–specific mortality and overall mortality compared with men without T2DM (13). Proposed mechanisms linking T2DM with prostate cancer diagnosis and outcomes include alterations in androgen receptor availability, lowered testosterone levels, and insulin resistance in men with T2DM (14–16). The role glycemic control plays in this complex relationship between T2DM and prostate cancer outcomes is not well known.

In this study, we sought to evaluate the association of glycemic control over time with prostate cancer–specific mortality in a large cohort of veterans with T2DM. A potential relationship between glycemic monitoring goals and significant prostate cancer outcomes can provide critical information to improve care delivery for patients with coincident T2DM and prostate cancer. Because glycemic control is not static, we examined this relationship using a time-dependent measure of glucose control. Also, given the significant racial disparities seen in both T2DM and prostate cancer outcomes, we stratified our analyses by race and ethnicity to determine the stability of the associations across racial and ethnic groups (17, 18).

## Materials and Methods

### Study design and data source

This was a retrospective cohort study of national clinical and administrative data in adult male veterans with T2DM using the Veterans Health Administration (VHA) Corporate Data Warehouse (CDW). Multiple clinical and administrative files from the CDW were linked to create the unique cohort dataset. The Veterans Health Information Systems and Technology Architecture was the primary source for the CDW data extracts, which included demographic, laboratory, diagnosis code, and prescription data embedded in outpatient visits, outpatient pharmacy encounters, and inpatient admission domains. In addition, we obtained VA Informatics and Computing Infrastructure oncology information from CDW raw data. We included the National Death Index (NDI) dataset to determine the cause of death during the follow-up period (January 1, 2010, to December 31, 2019) and the prostate cancer data core established within the VA Informatics and Computing Infrastructure. We obtained administrative and clinical datasets for baseline information (January 1, 2008, to December 31, 2009) followed longitudinally from January 1, 2010, until December 31, 2019 (or death or loss to follow-up). We used Structured Query Language to retrieve datasets from multiple CDW tables and linked them using patient scrambled social security numbers. This article represents the views of the authors and not those of the VHA or of the Medical University of South Carolina. The Institutional Review Board at the Ralph H. Johnson VA Medical Center approved this study.

### Patient cohort

Using a previously validated algorithm through the VA Health Equity and Rural Outreach Innovation Center, male veterans with T2DM were identified by the presence of two or more International Classification of Diseases, Ninth Revision, Clinical Modification (ICD-9-CM: 250, 357.2, 362.0, and 366.41) or ICD-10-CM (E08.0–E09.9, E11.00–E11.9, and E13.0–E13.9) codes or a prescription for outpatient diabetes medication, including insulin (VA drug class code HS501), oral hypoglycemic agents (VA drug class code HS 502), and pramlintide (VA drug class code HS 509) between January 1, 2008, and December 31, 2019 (19, 20). Veterans diagnosed or receiving diabetes medication during the baseline period from January 1, 2008, to December 31, 2009, were included in the retrospective cohort. We excluded veterans that were less than 45 years old on January 1, 2010; veterans with prevalent cancer during January 1, 2000, to December 31, 2009; and veterans with a single follow-up year during January 1, 2010, to December 31, 2019. Cancer diagnoses were identified using ICD-9-CM and ICD-10-CM codes (Supplementary Table S1).

### Exposure

The primary exposure was time-updated hemoglobin A1c (HbA1c) values measured during the follow-up period of January 1, 2010, to December 31, 2019. We measured HbA1c as a continuous value and aggregated by year and classified into three categories: <7.0%, 7.0% to 8.0%, and ≥8.0% for the period from 2010 to 2019. For patients with more than one HbA1c in a given year, the means of the HbA1c values in that year were calculated. Variable categories were chosen based on recommended HbA1c target ranges for intensive (HbA1c <7%) and modified (7 < HbA1c < 8%) glycemic control presented in the VA/Department of Defense (DoD) Clinical Practice Guidelines for the Management of Type 2 Diabetes Mellitus (21). The HbA1c >8% category reflected veterans with uncontrolled T2DM and hyperglycemia.

### Study outcomes

The primary outcome was prostate cancer mortality during the follow-up period from 2010 to 2019. Through collaboration with VA and DoD partners, the joint VA–DoD Mortality Data Repository has released the NDI records for all known veterans, service members, and VHA users who died between January 1, 1979, and December 31, 2020. The provided search file was matched with all deaths in the Mortality Data Repository database from calendar years 2010 to 2019 based on the social security number. Date and cause of death information were identified for 327,560 veterans. The underlying causes of death for the NDI are coded using ICD-10-CM codes. Veterans were followed from the date of entry into the cohort until the date of death or the end of the observation period (22).

### Covariates

Demographic variables of age, race, and ethnicity were collected from the CDW. Age was treated as a continuous variable. Race and ethnicity data are self-reported by veterans and entered into the electronic health record. Race and ethnicity were categorized as non-Hispanic White (NHW), non-Hispanic Black (NHB), Hispanic, other, and missing. Other variables of interest included marital status (married, unmarried, and missing), residence (rural, urban, and missing), yearly primary care visits (continuous), service-connected disability status (yes or no), and the number of comorbidities (continuous) identified by Elixhauser coding algorithms (23). Rurality (rural vs. urban) was defined by rural–urban commuting area codes using the definitions supported by the VA Office of Rural Health based on resident zip code (24). Obesity was a categorical variable determined by body mass index (BMI) at baseline and categorized as obese if BMI ≥30 kg/m^2^ or nonobese if BMI <30 kg/m^2^. Prescriptions of insulin and oral glucose-lowering agents were identified during the study period and treated as binary variables (yes/no). For the competing risk regression models, the T2DM treatment variable was a categorical variable which included no medication, oral agents only, insulin only, and a combination of oral medications and insulin (20). Prescription of statin as a binary variable (yes/no) was created using VA drug class code CV350 that included atorvastatin, fluvastatin, lovastatin, pitavastatin, pravastatin, and simvastatin.

### Statistical analysis

Descriptive statistics for measured variables were computed to present the cohort characteristics overall and stratified by HbA1c category. Demographic and clinical characteristics were summarized and compared using proportions and *χ*^2^ tests for categorical variables and means with one-way ANOVA for continuous variables. We analyzed time until death, measured in years. Patients who were still alive or lost to follow-up were right-censored.

Cause-specific competing risk models were fitted in the survival analysis in which the interest lies in the probability of death from prostate cancer as the primary cause. Competing risks occur when patients are at risk of more than one mutually exclusive event such as death from prostate cancer and other different causes (25). Death from any other cause was considered a competing event. Unadjusted and adjusted models with the time-updated exposure were fitted to estimate the risk of prostate cancer mortality associated with HbA1c using flexible parametric modeling (stpm2 in Stata version 18; ref. 25). Sequential procedures were fitted to estimate the flexible parametric modeling using the following: (i) an unadjusted model; (ii) a model adjusted for demographic variables (age, race/ethnicity, marital status, location of residence, and service-connected disability); (iii) a model adjusted for demographic and clinical variables (annual primary care visit, Elixhauser comorbidities, and obesity); and (iv) a model adjusted for demographic, clinical, and treatment variables (statin prescription and glucose-lowering treatment). In the final adjusted model, we also tested the interaction of statin prescription and T2DM treatment. We developed models for the overall cohort and then stratified the analysis by race and ethnicity comparing the three cumulative incidence function curves of HbA1c groups for NHW, NHB, Hispanic, and other categories. The number of subjects with events was estimated using stdescribe of Stata (26, 27). HRs and associated 95% confidence intervals (95% CI) were estimated from unadjusted and adjusted models. Missing data for HbA1c follow-up values were imputed using the last observation carried forward method. Akaike information criterion and Bayesian information criterion were used to assess the goodness of fit of different models. The Weibull model with a lower Akaike information criterion and Bayesian information criterion was preferred over the other alternative models (log-logistic and log-normal; Supplementary Table S2).

We performed a sensitivity analysis, in which only veterans with stable HbA1c variables (e.g., same HbA1c category) throughout follow-up were included. In addition, the incidence rates (IR) and associated 95% CIs were estimated for the association between time-updated glycemic control and prostate cancer mortality for the entire cohort and stratified by race/ethnicity group. All analyses were conducted using Stata version 18 (RRID: SCR_012763).

### Data availability

The data generated in this study are available upon request from the corresponding author.

## Results

**Figure 1:**
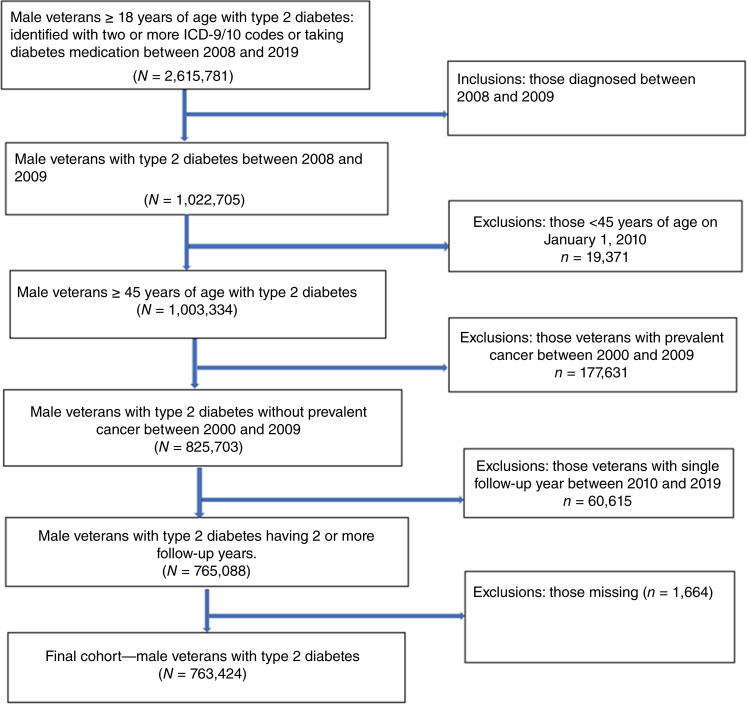
Consort diagram for creation of the veterans with T2DM cohort.

A total of 763,424 veterans with T2DM were included in the study and followed for a mean 5.1 years of follow-up (Fig. 1). Of the cohort, 294,913 (39%) had a baseline HbA1c <7.0%, 193,395 (25%) had a baseline HbA1c 7.0% to 8.0%, 162,050 (21%) a baseline HbA1c >8.0%, and the remaining 113,066 (15%) were missing a baseline HbA1c (Table 1). The mean age of veterans in the cohort at baseline was 66 years, and the majority were NHW (75%), married (60%), and resided in urban areas (62%). Cohort veterans had a mean of 3 Elixhauser comorbidities, a 50.4% prevalence of obesity (BMI ≥30 kg/m^2^), and attended an average of 4.3 primary care visits per year. During the baseline period, 84% of cohort patients were prescribed oral diabetes medication, 33% were prescribed insulin, and 79% were prescribed statin medication.

**Table 1:** Demographic and clinical characteristics at baseline among male veterans with type 2 diabetes in the cohort

*N* (%)	Total	A1c <7%	A1c 7%–8%	A1c >8%	Missing	*P* value
	763,424 (100)	294,913 (38.6)	193,395 (25.3)	162,050 (21.2)	113,066 (14.8)	
Mean age (SD)	65.6 (9.9)	66.6 (9.7)	65.9 (9.5)	62.1 (8.9)	67.7 (10.9)	<0.001*
Missing	2	0	0	0	0	​
Race/ethnicity, %	​	​	​	​	​	​
NHW	74.6	78.3	75.9	65.8	75.4	<0.001^†^
NHB	15.9	14.0	14.6	21.3	15.2	​
Hispanic	6.4	4.9	6.7	9.4	5.4	​
Other	2.7	2.4	2.6	2.9	3.4	​
Missing	0.4	0.3	0.3	0.6	0.5	​
Marital status, %	​	​	​	​	​	​
Unmarried	40.2	39.1	38.6	45.5	38.3	<0.001^†^
Married	59.7	60.8	61.3	54.5	61.6	​
Missing	0.08	0.07	0.07	0.06	0.1	​
Location of residence, %	​	​	​	​	​	​
Urban	61.6	60.2	60.6	63.5	63.9	<0.001^†^
Rural	38.2	39.6	39.2	36.3	35.5	​
Missing	0.2	0.2	0.2	0.2	0.6	​
Mean annual primary care visits (SD)	4.3 (4.2)	4.3 (4.3)	4.5 (4.2)	4.9 (4.5)	3.2 (3.4)	<0.001*
Service-connected disability (≥50%), %	​	​	​	​	​	​
No	86.1	85.5	85.9	85.2	89.2	<0.001^†^
Yes	13.9	14.5	14.1	14.8	10.8	​
Elixhauser comorbidities, mean (SD)	3.0 (1.9)	3.1 (1.9)	3.1 (1.9)	3.2 (1.8)	2.3 (1.8)	<0.01*
Obesity (BMI ≥30 kg/m^2^)	​	​	​	​	​	​
Yes	49.6	51.3	46.7	43.3	58.8	<0.001^†^
No	50.4	48.7	53.3	56.7	41.2	​
Statin use, %	​	​	​	​	​	​
No	21.2	20.4	17.7	18.4	33.2	<0.001^†^
Yes	78.8	79.6	82.3	81.6	66.8	​
Insulin use, %	​	​	​	​	​	​
No	66.6	84.1	62.2	35.2	73.1	<0.001^†^
Yes	33.4	15.9	37.8	64.8	26.9	​
Oral diabetes medication use, %	​	​	​	​	​	​
No	16.2	13.9	14.0	17.5	24.1	<0.001^†^
Yes	83.8	86.1	86.0	82.5	75.9	​

Annual primary care visit, service-connected disability, Elixhauser scores, obesity, statin use, insulin use, and oral medication use are for baseline 2008 to 2009.

*ANOVA test.

^†^ *χ* ^2^ test.

**Figure 2:**
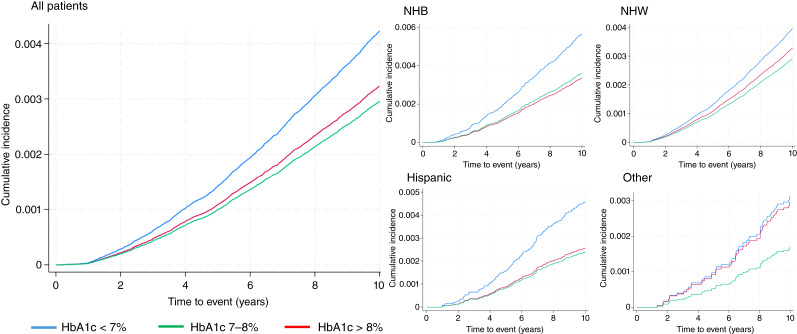
Cause-specific cumulative incidence function for HbA1c control based on Fine and Gray regression models for the overall cohort and stratified by race and ethnicity.

**Figure 3:**
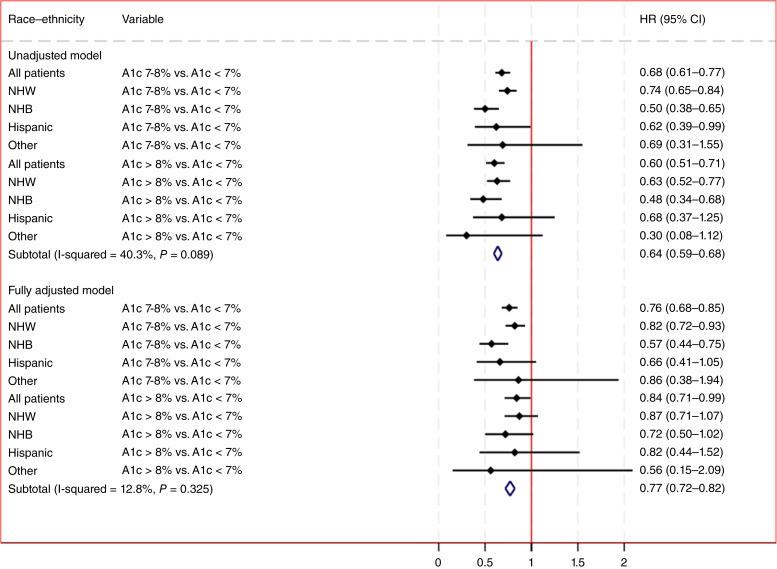
Estimated HRs from the unadjusted and adjusted cause-specific competing risk models evaluating the association between time-updated glycemic control and prostate cancer mortality for the entire cohort and stratified by race/ethnicity group.

There were 2,723 prostate cancer deaths during the follow-up period, with 1,346 (49%) occurring in individuals with HbA1c <7%, 730 (27%) in veterans with HbA1c 7% to 8%, and 647 (24%) in those with HbA1c >8%. Table 2 depicts the HR for cause-specific risk models with time-updated glycemic control for unadjusted and adjusted models for the overall cohort. For the unadjusted model, HbA1c 7% to 8% (HR, 0.68; 95% CI 0.61–0.77) and HbA1c >8% (HR, 0.60; 95% CI, 0.51–0.71) were associated with a lower risk of prostate cancer mortality compared with HbA1c <7 (Figs. 2 and 3).

**Table 2:** Cause-specific competing risk models for the association between time-updated glycemic control and prostate cancer mortality in male veterans with type 2 diabetes (with TVC)

​	HRs and 95% CIs using flexible parametric models—stpm2 in Stata							
Variables	Model 0		Model 1		Model 2		Model 3	
*N*	*n* = 763,411		*n* = 757,872		*n* = 757,872		*n* = 757,872	
	HR (95% CI)	*P* value	HR (95% CI)	*P* value	HR (95% CI)	*P* value	HR (95% CI)	*P* value
Exposure	​	​	​	​	​	​	​	​
A1c <7% (ref.)	1 (ref.)	​	1 (ref.)	—	1 (ref.)	​	1 (ref.)	—
A1c 7%–8%	0.68 (0.61–0.77)	<0.001	0.77 (0.68–0.85)	<0.001	0.77 (0.69–0.86)	<0.001	0.76 (0.68–0.85)	<0.001
A1c >8%	0.60 (0.51–0.71)	<0.001	0.85 (0.72–0.99)	0.044	0.845 (0.72–0.99)	0.042	0.84 (0.71–0.99)	0.037
Demographic variables	​	​	​	​	​	​	​	​
NHW (ref.)	​	​	1 (ref.)	—	1 (ref.)	​	1 (ref.)	—
NHB	​	​	1.92 (1.74–2.12)	<0.001	1.92 (1.74–2.12)	<0.001	1.91 (1.73–2.11)	<0.001
Hispanic	​	​	1.04 (0.88–1.23)	0.628	1.05 (0.89–1.24)	0.563	1.06 (0.90–1.25)	0.515
Other	​	​	0.96 (0.73–1.25)	0.747	0.97 (0.74–1.26)	0.803	0.97 (0.74–1.27)	0.815
Age (continuous)	​	​	1.12 (1.11–1.12)	<0.001	1.12 (1.11–1.12)	<0.001	1.11 (1.11–1.12)	<0.001
Unmarried (ref.)	​	​	1 (ref.)	—	1 (ref.)	​	1 (ref.)	—
Married	​	​	0.91 (0.84–0.98)	0.017	0.91 (0.84–0.99)	0.020	0.90 (0.84–0.98)	0.010
Urban (ref)	​	​	1 (ref.)	—	1 (ref.)	​	1 (ref.)	—
Rural	​	​	1.15 (1.06–1.24)	0.001	1.15 (1.06–1.24)	0.001	1.15 (1.07–1.25)	<0.001
Service-connected disability < 50% (ref.)	​	​	1 (ref.)	—	1 (ref.)	​	1 (ref.)	—
Service-connected disability ≥ 50%	​	​	0.80 (0.70–0.92)	0.002	0.79 (0.69–0.91)	0.001	0.80 (0.69–0.92)	0.001
Clinical variables	​	​	​	​	​	​	​	​
Primary care visits per year	​	​	​	​	1.00 (0.99–1.01)	0.796	1.00 (0.99–1.01)	0.997
Elixhauser comorbidity	​	​	​	​	1.02 (0.99–1.04)	0.207	1.02 (0.99–1.04)	0.223
Obesity (BMI ≥30 kg/m^2^)	​	​	​	​	1.09 (1.00–1.18)	0.044	1.09 (1.01–1.19)	0.033
Treatment variables	​	​	​	​	​	​	​	​
No statin use (ref.)	​	​	​	​	​	​	1 (ref.)	​
Statin use	​	​	​	​	​	​	0.74 (0.67–0.82)	<0.001
T2DM treatment	​	​	​	​	​	​	​	​
No medication (ref.)	​	​	​	​	​	​	1 (ref.)	—
Oral medication use only	​	​	​	​	​	​	0.93 (0.81–1.06)	0.275
Insulin use only	​	​	​	​	​	​	1.23 (1.05–1.45)	0.011
Both insulin and oral medication use	​	​	​	​	​	​	0.93 (0.80–1.07)	0.306

Abbreviations: Stpm, specific-time parametric modeling; TVC, time varying covariate.

Model 0 = unadjusted model.

Model 1 = model 0 + demographic variables (age, race/ethnicity. marital status, location of residence, and service-connected disability).

Model 2 = model 1 + clinical variables (annual primary care visit + Elixhauser comorbidity + obesity).

Model 3 = model 2 + treatment variables [statin use and T2DM treatments (oral medication, insulin, or both)].

In the fully adjusted model incorporating demographic, clinical, and treatment variables, elevated HbA1c 7% to 8% (HR, 0.76; 95% CI, 0.68–0.85) and HbA1c >8% (HR, 0.85; 95% CI, 0.71–0.99) were associated with a lower risk of mortality from prostate cancer compared with HbA1c <7%. The sequentially adjusted models demonstrate consistent estimates for the association between HbA1c categories and risk of prostate cancer mortality across the entire cohort. Additionally, in the fully adjusted model, patients identified as NHB had a higher risk of prostate cancer mortality (HR, 1.91; 95% CI, 1.73–2.11) compared with NHW veterans. Hispanic (HR, 1.06; 95% CI, 0.90–1.25) and other (HR, 0.97; 95% CI, 0.74–1.25) categories of race and ethnicity were not associated with prostate cancer mortality risk compared with NHW veterans. Married veterans (HR, 0.90; 95% CI, 0.84–0.98) had a lower risk of mortality, whereas rural veterans (HR, 1.15; 95% CI, 1.07–1.25) had a higher risk of mortality from prostate cancer. From a T2DM treatment perspective, statin prescriptions were associated with a lower risk of prostate cancer mortality (HR, 0.74; 95% CI, 0.67–0.82). Insulin use without oral medications for glycemic management was associated with a higher risk of prostate cancer mortality (HR, 1.23; 95% CI, 1.05–1.45), whereas oral medication prescriptions and combinations of insulin and oral glucose-lowering agents were not associated with significant differences in mortality, compared with patients without antihyperglycemic medications.

Figure 2 shows the cause-specific cumulative incidence function curves stratified by race and ethnicity for HbA1c control with estimated rates of cumulative prostate cancer mortality over time. The unadjusted cause-specific competing risk model for veterans identified as NHW demonstrated that HbA1c 7% to 8% (HR, 0.74; 95% CI, 0.65–0.84) and HbA1c >8% (HR, 0.63; 95% CI, 0.52–0.77) were associated with a lower risk of prostate cancer mortality compared with veterans with HbA1c <7% (Table 3). After adjusting for all of the available variables, HbA1c 7% to 8% (HR, 0.82; 95% CI, 0.72–0.93) remained associated with a lower risk of mortality, whereas HbA1c >8% (HR, 0.87; 95% CI, 0.71–1.07) was no longer associated with the outcome. For veterans identified as NHB, the unadjusted models demonstrated a lower risk of prostate cancer mortality for veterans with HbA1c 7% to 8% (HR, 0.50; 95% CI, 0.38–0.65) and HbA1c >8% (HR, 0.48; 95% CI, 0.34–0.68) compared with patients with HbA1c <7%. The fully adjusted Cox regression model for NHB veterans demonstrated that HbA1c 7% to 8% (HR, 0.57; 95% CI, 0.44–0.75) was associated with a lower risk of prostate cancer mortality, but HbA1c >8% (HR, 0.72; 95% CI, 0.50–1.02) was not significantly associated with the outcome. There were no significant associations between HbA1c 7% to 8% or HbA1c >8% with prostate cancer mortality in the fully adjusted competing risk models for veterans identified as Hispanic or of other race and ethnicity. Measures of model performance and details of the race- and ethnicity-stratified cause-specific competing risk models are in Supplementary Tables S2 and S3A–S3D.

**Table 3:** Cause-specific competing risk models for the association between time-updated glycemic control and prostate cancer mortality in male veterans with type 2 diabetes stratified by race and ethnicity

​	HRs and 95% CIs using flexible parametric models—stpm2 in Stata				
Variables	Events	Model 0	Model 1	Model 2	Model 3
*N*		*n* = 569,691	*n* = 568,754	*n* = 568,754	*n* = 568,754
		HR (95% CI)	HR (95% CI)	HR (95% CI)	HR (95% CI)
NHW	​	​	​	​	​
A1c <7% (ref.)	957	1 (ref.)	1 (ref.)	1 (ref.)	1 (ref.)
A1c 7%–8%	570	0.74 (0.65–0.84)	0.82 (0.72–0.93)	0.82 (0.72–0.93)	0.82 (0.72–0.93)
A1c >8%	448	0.63 (0.52–0.77)	0.88 (0.72–1.07)	0.88 (0.72–1.07)	0.87 (0.71–1.07)
NHB	​	​	​	​	​
A1c <7% (ref.)	283	1 (ref.)	1 (ref.)	1 (ref.)	1 (ref.)
A1c 7%–8%	104	0.50 (0.38–0.65)	0.57 (0.44–0.75)	0.58 (0.44–0.75)	0.57 (0.44–0.75)
A1c >8%	143	0.48 (0.34–0.68)	0.73 (0.52–1.03)	0.74 (0.52–1.04)	0.72 (0.50–1.02)
Hispanic	​	​	​	​	​
A1c <7% (ref.)	78	1 (ref.)	1 (ref.)	1 (ref.)	1 (ref.)
A1c 7%–8%	36	0.62 (0.39–0.99)	0.67 (0.42–1.07)	0.68 (0.43–1.08)	0.66 (0.41–1.05)
A1c >8%	42	0.68 (0.37–1.25)	0.88 (0.49–1.61)	0.89 (0.49–1.63)	0.82 (0.44–1.52)
Other	​	​	​	​	​
A1c <7% (ref.)	26	1 (ref.)	1 (ref.)	1 (ref.)	1 (ref.)
A1c 7%–8%	18	0.69 (0.31–1.55)	0.78 (0.35–1.73)	0.78 (0.35–1.74)	0.86 (0.39–1.94)
A1c >8%	11	0.30 (0.08–1.12)	0.43 (0.12–1.60)	0.43 (0.12–1.60)	0.56 (0.15–2.09)

Abbreviation: Stpm, specific-time parametric modeling.

Model 0 = unadjusted model.

Model 1 = model 0 + demographic variables (age, race/ethnicity. marital status, location of residence, and service-connected disability).

Model 2 = model 1 + clinical variables (annual primary care visit + Elixhauser comorbidity + obesity).

Model 3 = model 2 + treatment variables [statin use and T2DM treatments (oral medication, insulin, or both)].

Full models in Supplementary Table S3A–S3D.

The sensitivity analysis of veterans with a stable HbA1c category throughout the duration of follow-up included 226,161 unique veterans. In the unadjusted cause-specific competing risk model, HbA1c 7% to 8% (HR, 1.88; 95% CI, 1.55–2.28) was associated with an increased risk of prostate cancer mortality compared with HbA1c <7%, whereas HbA1c >8% (HR, 0.77; 95% CI, 0.61–0.99) was associated with a lower risk of prostate cancer mortality. After adjusting for demographic, clinical, and T2DM treatment variables, HbA1c 7% to 8% (HR, 1.45; 95% CI, 1.19–1.76) was associated with an increased hazard of prostate cancer mortality whereas HbA1c >8% (1.19; 95% CI, 0.93–1.53) had no significant association with the primary outcome, compared with HbA1c <7% (Supplementary Table S4).

The estimated IR per 1,000 veterans for the association between time-updated glycemic control and prostate cancer mortality are presented in Fig. 2 and Supplementary Table S5. For the IR, HbA1c <7% (IR, 0.54; 95% CI, 0.51–0.56), HbA1c 7% to 8% (IR, 0.39; 95% CI, 0.37–0.42), and HbA1c >8% (IR, 0.36; 95% CI, 0.34–0.39) continuously declined from HbA1c 7% to HbA1c >8%, and similar patterns were observed for NHW, Hispanic, and other race/ethnicity veterans. For NHB veterans, the IR declined from HbA1c <7% (IR, 0.69; 95% CI, 0.61–0.77) to HbA1c 7%–8% (IR, 0.38; 95% CI, 0.32–0.47) and then slightly increased to HbA1c >8% (IR, 0.41; 95% CI, 0.35–0.49).

## Discussion

In this large, longitudinal cohort study of male veterans with T2DM, time-dependent measures of glycemic control were inversely associated with cause-specific prostate cancer mortality. Time-dependent measures of HbA1c >8% (HR, 0.84; 95% CI, 0.71–0.99) and HbA1c measures between 7% and 8% (HR, 0.76; 95% CI, 0.68–0.85) were associated with a significantly lower hazard of prostate cancer mortality compared with HbA1c measures <7% in the fully adjusted models. This association of time-dependent measures of HbA1c with prostate cancer mortality was attenuated in the analyses stratified by race and ethnicity. There was no significant association of HbA1c >8% with prostate cancer deaths across all racial and ethnic groups in the stratified analysis. HbA1c measures between 7% and 8% were still associated with a lower risk of prostate cancer mortality in NHW (HR, 0.82; 95% CI, 0.72–0.93) and NHB (HR, 0.57; 95% CI, 0.44–0.75) veterans compared with HbA1c <7%.

Hyperglycemia has previously been linked to increased cancer incidence, heightened cancer aggressiveness, and poor cancer-related health outcomes. Mechanistically, it is thought that prolonged elevations of serum glucose contribute to worsen cancer prognosis through deregulation of cellular energetics, promotion of cell proliferation, increased metastatic potential, stimulation of angiogenesis, and incurrence of cell death resistance (28). The negative impacts of hyperglycemia on cancer survival outcomes have been seen in gastric, colorectal, breast, cervical, and blood-based cancers (29–32). However, the relationship between hyperglycemia and prostate cancer is less well understood. Whereas previous studies have demonstrated an inverse association between T2DM and prostate cancer incidence, hyperglycemia has been linked with detection of higher-grade prostate cancers and an increased risk of prostate cancer metastasis (33–35). In this study, we demonstrated a lower risk of prostate cancer–specific mortality with HbA1c measures of 7% to 8% and >8% compared with HbA1c <7% using time-varying measures of glycemic control in competing risk models. Additionally, this is among the first studies to investigate the persistence of this relationship across racial and ethnic groups. Potential mechanisms for hyperglycemia conferring some protective benefit for prostate cancer–specific mortality in patients with T2DM include lower levels of circulating testosterone, therapeutic benefits from T2DM-directed therapies, and the potentially protective effect of T2DM-related endothelial damage on prostate cancer progression (36, 37). Clinically, these findings may present an opportunity to improve prostate cancer care if this relationship is found in prospective studies. Liberalizing therapeutic glycemic targets to 7% to 8% in patients at risk, or with prostate cancer, could be integrated into primary care, similar to how therapeutic targets are modified for patients with T2DM of advancing age (38). However, it is also necessary to consider that this observed relationship between time-dependent measures of HbA1c and prostate cancer mortality could be influenced by unmeasured biases. For instance, patients with higher HbA1c values may receive a higher intensity of care which yields a longevity benefit. Because the HbA1c exposure in these analyses was time-dependent, the HbA1c <7% values may capture episodes HbA1c variability and hypoglycemia, which have been associated with poor cardiovascular and cancer outcomes (39, 40). The sensitivity analysis of only veterans with stable measures of HbA1c during follow-up supports this hypothesis, as HbA1c of 7% to 8% was associated with an increased hazard of prostate mortality (compared with HbA1c <7%) and HbA1c >8% was not associated with the primary outcome in the subset. Thus, veterans with HbA1c measures that change categories over time seem to be driving the observed results in the full sample.

Future work needs to explore the role of plausible mechanism and confounding variables in this relationship. Testosterone levels, which are not routinely measured in usual patient care and were not available for all veterans in our dataset, would be beneficial for further investigating the potentially protective role of higher HbA1c measures on prostate cancer mortality. Medications, specifically exogenous insulin, statins, and in the future, glucagon-like peptide 1 receptor agonists and sodium–glucose cotransporter 2 inhibitors, may modify the relationship between T2DM, glycemic control, and prostate cancer (41). In the fully adjusted model for the full cohort in this study, prescription of a statin medication was associated with a lower risk of prostate cancer mortality (HR, 0.74; 95% CI, 0.67–0.82), whereas the use of exogenous insulin only (with no oral, antihyperglycemic medication) was associated with an increased risk of prostate cancer death (HR, 1.23; 95% CI, 1.05–1.45). A meta-analysis examining the relationship between statin exposure and prostate cancer prognosis aligns with these findings, demonstrating a reduced incidence and better prognosis of prostate cancer with statin use (42). The relationship between insulin use and prostate cancer outcomes is less clear and requires further study (43). These medication variables were treated as time-fixed in our study, which does limit the interpretation of their relationship with prostate cancer mortality. Additionally, this study did not include variables for prostate cancer stage, receipt of prostate cancer therapy, or response to therapy in the analysis. It is possible that cancer stage and treatment are related to HbA1c values, as well as prostate cancer outcomes. Advanced disease and androgen deprivation therapy could result in weight loss, which might reduce HbA1c measures and affect prostate cancer outcomes (44). A better understanding of the association of circulating androgen levels, diabetes pharmacotherapy exposure, prostate cancer staging, and prostate cancer treatment response could provide greater clarity into the relationship between glycemic control and prostate cancer mortality outcomes.

There are limitations in this study. First, this is an observation study, so no causal link between glycemic measures and prostate cancer mortality can be determined. Additionally, this is exclusively a study of veterans, and findings may not be generalizable to all US males. However, the VA is the largest single-payer, equal-access healthcare system in the United States, which allowed us to observe equitable treatment characteristics regarding follow-up visit time and medication usage. Also, this study relies upon diagnosis codes and would not capture veterans with T2DM that are without a formal diagnosis (45). Furthermore, variables for prostate cancer stage, receipt of therapy, and therapeutic response are not available. As with any large dataset, there is the threat of unmeasured confounding and omitted variables. Specifically, the dataset has limited socioeconomic information including education and income, which are significant social determinants of health. Observed differences in the results stratified by race and ethnicity may be related to an unmeasured socioeconomic variable that differed between groups. Future work that more comprehensively incorporates social determinant of health variables and mediators is needed.

In this large, retrospective study of male veterans with T2DM, we observed an inverse association between degrees of glycemic control and prostate cancer mortality. Similar findings were observed in stratified analyses for veterans identified as NHW and NHB.

## Supplementary Material

Supplementary Table S1 Site-specific cancer diagnosis codes.

Supplementary Table S2 Model selection table from the flexible parametric models based on: -2logL, Akaike Information Criterion (AIC), Bayesian Information Criterion (BIC) and C-statistic.

Supplementary Table S3a Cause-specific competing risk models for the association between time-updated glycemic control and prostate cancer mortality in male veterans with type-2 diabetes among Non-Hispanic White (with TVC).

Supplementary Table S3b Cause-specific competing risk models for the association between time-updated glycemic control and prostate cancer mortality in male veterans with type-2 diabetes among Non-Hispanic Black (with TVC).

Supplementary Table S3c Cause-specific competing risk models for the association between time-updated glycemic control and prostate cancer mortality in male veterans with type-2 diabetes among Hispanic (with TVC).

Supplementary Table S3d Cause-specific competing risk models for the association between time-updated glycemic control and prostate cancer mortality in male veterans with type-2 diabetes among Other

Supplementary Table S4 Cause-specific competing risk models for the association between time-updated glycemic control and prostate cancer mortality in male Veterans with type-2 diabetes and a stable hemoglobin A1c category throughout follow-up.

Supplementary Table S5 Estimated incidence rates (per 1,000) for the association between time-updated glycemic control and prostate cancer mortality for the entire cohort and stratified by race/ethnicity group.
