# Supplementary material for: Glycemic Control and Prostate Cancer Mortality Risk in Veterans with Type 2 Diabetes Mellitus
Source: Cancer Res Commun. 2025 Aug 1;5(8):1256–65. doi: 10.1158/2767-9764.CRC-25-0037 (PMC12314478; doi:10.1158/2767-9764.CRC-25-0037)
Supplement: Supplementary Table S1 — Site-specific cancer diagnosis codes. [file crc-25-0037_supplementary_table_s1_suppst1.pdf]

Supplementary Table S1. Site-specific cancer diagnosis codes.

Cancer Site	ICD-9 diagnosis code	ICD-10 diagnosis code
Head and neck	140.0 – 149.9	C00.0 – C14.8
Colon	153.0 – 153.9	C18.0 – C18.9
Rectal	154.0 – 154.8	C20
Liver	155.0 – 155.2	C22.0 – C22.9
Pancreas	157.0 – 157.9	C25.0 – C25.9
Stomach	151.0 – 151.9	C16.0 – C16.9
Other digestive	150.0 – 150.9, 152.0 – 152.9, 156.0 – 156.9, 158.0 – 158.9, 159.0 – 159.9	C15.3 – C15.9, C17.0 – C17.9, C19, C21.0 – C21.8, C23, C24.0 - C24.9, C26.0 – C26.9
Lung/bronchus	162.0 – 162.9	C34.00 – C34.92
Other respiratory	160.0 – 160.9, 161.0 – 161.9, 163.0 – 163.9, 164.0 – 164.9, 165.0 – 165.9	C30.0 – C33, C37 – C39.9
Hematopoietic/lymphatic	196.0 – 196.9	C81.00 – C96.0
Melanoma	172.0 – 172.9	C43.0 – C43.9
Breast (male)	175.0, 175.9	C50.021 – C50.029, C50.121 – CC50.129, C50.221 – C50.229, C50.321 – C50.329, C50.421 – C50.429, C50.521 – C50.529, C50.621 – C50.629, C50.821 – C50.829, C50,921 – C50.929
Prostate	185	C61
Bladder	188.0 – 188.9	C67.0 – C67.9
Kidney	189.0 – 189.9	C64.1 – C64.9
Thyroid	193	C73
Brain and other CNS	191.0 – 191.9, 192.0 – 192.9	C70.0 – C72.9,
Unspecified site	195.0 – 195.8, 199.0 – 199.2	C40 – C41, C44.00 – C44.99

Other	170.0 – 171.9, 176.0 – 176.9, 186.0 – 187.9, 190.0 – 190.9, 194.0 – 194.9	C45.0 – C49.9, C49.A0 – C49.A9, C60.0 – C60.9, C62.00 – C62.92, C63.00 – C63.9, C65.1 – C66.9, C68.0 – C68.9, C69.00 – C69.92, C74.00 – C76.8, C80.0 – C80.2

ICD=International Classification of Diseases.
