# Supplementary material for: Glycemic Control and Prostate Cancer Mortality Risk in Veterans with Type 2 Diabetes Mellitus
Source: Cancer Res Commun. 2025 Aug 1;5(8):1256–65. doi: 10.1158/2767-9764.CRC-25-0037 (PMC12314478; doi:10.1158/2767-9764.CRC-25-0037)
Supplement: Supplementary Table S2 — Model selection table from the flexible parametric models based on: -2logL, Akaike Information Criterion (AIC), Bayesian Information Criterion (BIC) and C-statistic. [file crc-25-0037_supplementary_table_s2_suppst2.pdf]

Supplementary Table S2: Model selection table from the flexible parametric models based on: -2logL, Akaike Information Criterion (AIC), Bayesian Information Criterion (BIC) and C-statistic.

Group	Statistics	Model 0	Model 1	Model 2	Model 3
**All patient**					
	**-2log-likelihood**	37,249	34,344	34,336	34,278
	**Akaike Information Criterion (AIC)**	37,259	34,368	34,365	34,312
	**Bayesian Information Criterion (BIC)**	37,327	34,532	34,571	34,571
	**C-statistic**	0.545	0.748	0.748	0.750
**Non-Hispanic White**					
	**-2log-likelihood**	27,015	25,071	25,064	25,029
	**Akaike Information Criterion (AIC)**	27,026	25,090	25,088	25,061
	**Bayesian Information Criterion (BIC)**	27,092	25,210	25,248	25,275
	**C-statistic**	0.545	0.741	0.741	0.742
**Non-Hispanic Black**					
	**-2log-likelihood**	7,105	6,506	6,499	6,478
	**Akaike Information Criterion (AIC)**	7,115	6,524	6,524	6,510
	**Bayesian Information Criterion (BIC)**	7,175	6,631	6,666	6,699
	**C-statistic**	0.569	0.766	0.767	0.774
**Hispanic**					
	**-2log-likelihood**	2,188	2,022	2,020	2,008
	**Akaike Information Criterion (AIC)**	2,199	2,042	2,044	2,040
	**Bayesian Information Criterion (BIC)**	2,253	2,140	2,176	2,216
	**C-statistic**	0.583	0.771	0.772	0.774
**Other**					
	**-2log-likelihood**	783	714	713	705
	**Akaike Information Criterion (AIC)**	793	732	737	737
	**Bayesian Information Criterion (BIC)**	844	822	858	897
	**C-statistic**	0.562	0.752	0.752	0.770

Model 0 = Unadjusted model

Model 1 = Model 0 + demographic variables (age, race/ethnicity. marital status, location of residence, service-connected disability).

Model 2 = Model 1 + clinical variables (annual primary care visit + Elixhauser comorbidity).

Model 3 = Model 2 + Obesity + treatment variable (statin use) + T2DM treatment
