# Supplementary material for: Glycemic Control and Prostate Cancer Mortality Risk in Veterans with Type 2 Diabetes Mellitus
Source: Cancer Res Commun. 2025 Aug 1;5(8):1256–65. doi: 10.1158/2767-9764.CRC-25-0037 (PMC12314478; doi:10.1158/2767-9764.CRC-25-0037)
Supplement: Supplementary Table S3a — Cause-specific competing risk models for the association between time-updated glycemic control and prostate cancer mortality in male veterans with type-2 diabetes among Non-Hispanic White (with TVC). [file crc-25-0037_supplementary_table_s3a_suppst3a.pdf]

Supplementary Table S3a. Cause-specific competing risk models for the association between time-updated glycemic control and prostate cancer mortality in male veterans with type-2 diabetes among Non-Hispanic White (with TVC).

	Hazard Ratios (HR) and 95% Confidence Intervals (CIs) using Flexible Parametric Models-stpm2 in Stata											
**Variables**	**Model 0**			**Model 1**			**Model 2**			**Model 3**		
**N**	**n=569,691**			**n=568,754**			**n=568,754**			**n=568,754**		
	**Events**	**HR (95% CI)**	**p-value**	**Events**	**HR (95% CI)**	**p-value**	**Events**	**HR (95% CI)**	**p-value**	**Events**	**HR (95% CI)**	**p-value**
**Exposure**												
A1c < 7% (ref.)	957	1 (ref.)		957	1 (ref.)	-	957	1 (ref.)		957	1 (ref.)	-
A1c 7-8%	570	0.74 (0.65, 0.84)	<0.001	570	0.82 (0.72, 0.93)	0.002	570	0.82 (0.72, 0.93)	0.002	570	0.82 (0.72, 0.93)	0.003
A1c >8%	448	0.63 (0.52, 0.77)	<0.001	447	0.88 (0.72, 1.07)	0.200	447	0.88 (0.72, 1.07)	0.193	447	0.87 (0.71, 1.07)	0.183
**Demographic variables**												
Age (continuous)					1.12 (1.11, 1.12)	<0.001		1.12 (1.11, 1.12)	<0.001		1.12 (1.11, 1.12)	<0.001
Non-married (ref.)					1 (ref.)	-		1 (ref.)			1 (ref.)	-
Married					0.92 (0.84, 1.00)	0.058		0.92 (0.84, 1.01)	0.076		0.91 (0.83, 1.00)	0.045
Urban (ref)					1 (ref.)	-		1 (ref.)			1 (ref.)	-
Rural					1.13 (1.03, 1.23)	0.008		1.13 (1.03, 1.23)	0.008		1.13 (1.04, 1.24)	0.006
Service-connected disability <50% (ref.)					1 (ref.)	-		1 (ref.)			1 (ref.)	-
Service-connected disability >=50%					0.83 (0.70, 0.98)	0.026		0.82 (0.69, 0.97)	0.018		0.82 (0.70, 0.97)	0.021
**Clinical variables**												
Annual primary care visit (continuous)								1.01 (1.00, 1.02)	0.203		1.01 (1.00, 1.02)	0.141
Elixhauser comorbidity (continuous)								1.00 (0.97, 1.03)	0.802		1.01 (0.98, 1.03)	0.734

Obesity<br>(BMI ≥30 kg/m^2)								1.11 (1.01, 1.23)			1.12 (1.01, 1.23)	0.029
**Treatment variables**												
No statin use (ref.)											1 (ref.)	
Statin use											0.78 (0.69, 0.89)	<0.001
**T2DM Treatment**												
No medication (ref.)											1 (ref.)	-
Oral medication use only											0.92 (0.79, 1.07)	0.278
Insulin use only											1.18 (0.98, 1.43)	0.085
Both insulin and oral medication use											0.90 (0.76, 1.07)	0.231

Model 0 = Unadjusted model

Model 1 = Model 0 + demographic variables (age, race/ethnicity. marital status, location of residence, service-connected disability).

Model 2 = Model 1 + clinical variables (Annual primary care visit + Elixhauser comorbidity + Obesity).

Model 3 = Model 2 + treatment variable (statin use) + T2DM treatment
