# Supplementary material for: Glycemic Control and Prostate Cancer Mortality Risk in Veterans with Type 2 Diabetes Mellitus
Source: Cancer Res Commun. 2025 Aug 1;5(8):1256–65. doi: 10.1158/2767-9764.CRC-25-0037 (PMC12314478; doi:10.1158/2767-9764.CRC-25-0037)
Supplement: Supplementary Table S3b — Cause-specific competing risk models for the association between time-updated glycemic control and prostate cancer mortality in male veterans with type-2 diabetes among Non-Hispanic Black (with TVC). [file crc-25-0037_supplementary_table_s3b_suppst3b.pdf]

Supplementary Table S3b. Cause-specific competing risk models for the association between time-updated glycemic control and prostate cancer mortality in male veterans with type-2 diabetes among Non-Hispanic Black (with TVC).

	Hazard Ratios (HR) and 95% Confidence Intervals (CIs) using Flexible Parametric Models-stpm2 in Stata											
Variables	Model 0			Model 1			Model 2			Model 3		
N	n=121,150			n=120,868			n=120,868			n=120,868		
	Events	HR (95% CI)	p-value	Events	HR (95% CI)	p-value	Events	HR (95% CI)	p-value	Events	HR (95% CI)	p-value
**Exposure**												
A1c < 7% (ref.)	283	1 (ref.)		283	1 (ref.)	-	283	1 (ref.)		283	1 (ref.)	-
A1c 7-8%	104	0.50 (0.38, 0.65)	<0.001	104	0.57 (0.44, 0.75)	<0.001	104	0.58 (0.44, 0.75)	<.001	104	0.57 (0.44, 0.75)	<0.001
A1c >8%	143	0.48 (0.34, 0.68)	<0.001	143	0.73 (0.52, 1.03)	0.075	143	0.74 (0.52, 1.04)	0.082	143	0.72 (0.50, 1.02)	0.067
**Demographic variables**												
Age (continuous)					1.11 (1.10, 1.12)	<0.001		1.11 (1.10, 1.12)	<0.001		1.11 (1.10, 1.12)	<0.001
Non-married (ref.)					1 (ref.)	-		1 (ref.)			1 (ref.)	-
Married					0.90 (0.76, 1.07)	0.239		0.90 (0.76, 1.07)	0.225		0.90 (0.76, 1.07)	0.215
Urban (ref)					1 (ref.)	-		1 (ref.)			1 (ref.)	-
Rural					1.15 (0.94, 1.42)	0.184		1.14 (0.93, 1.41)	0.207		1.15 (0.93, 1.41)	0.195
Service-connected disability <50% (ref.)					1 (ref.)	-		1 (ref.)			1 (ref.)	-
Service-connected disability >=50%					0.77 (0.57, 1.03)	0.075		0.76 (0.57, 1.02)	0.066		0.75 (0.56, 1.01)	0.060
**Clinical variables**												
Annual primary care visit (continuous)								0.97 (0.94, 1.00)	0.021		0.97 (0.94, 1.00)	0.029

Elixhauser comorbidity (continuous)								1.04 (0.99, 1.09)	0.137		1.03 (0.98, 1.09)	0.205
Obesity (BMI ≥30 kg/m^2)								1.01 (0.84, 1.21)	0.919		1.02 (0.85, 1.23)	0.795
**Treatment variables**												
No statin use (ref.)											1 (ref.)	
Statin use											0.64 (0.50, 0.80)	<0.001
**T2DM Treatment**												
No medication (ref.)											1 (ref.)	-
Oral medication use only											0.92 (0.67, 1.27)	0.616
Insulin use only											1.30 (0.90, 1.89)	0.158
Both insulin and oral medication use											1.00 (0.71, 1.40)	0.977

Model 0 = Unadjusted model

Model 1 = Model 0 + demographic variables (age, race/ethnicity. marital status, location of residence, service-connected disability).

Model 2 = Model 1 + clinical variables (Annual primary care visit + Elixhauser comorbidity + Obesity).

Model 3 = Model 2 + treatment variable (statin use) + T2DM treatment
