# Supplementary material for: Glycemic Control and Prostate Cancer Mortality Risk in Veterans with Type 2 Diabetes Mellitus
Source: Cancer Res Commun. 2025 Aug 1;5(8):1256–65. doi: 10.1158/2767-9764.CRC-25-0037 (PMC12314478; doi:10.1158/2767-9764.CRC-25-0037)
Supplement: Supplementary Table S3c — Cause-specific competing risk models for the association between time-updated glycemic control and prostate cancer mortality in male veterans with type-2 diabetes among Hispanic (with TVC). [file crc-25-0037_supplementary_table_s3c_suppst3c.pdf]

Supplementary Table S3c. Cause-specific competing risk models for the association between time-updated glycemic control and prostate cancer mortality in male veterans with type-2 diabetes among Hispanic (with TVC).

	Hazard Ratios (HR) and 95% Confidence Intervals (CIs) using Flexible Parametric Models-stpm2 in Stata											
**Variables**	**Model 0**			**Model 1**			**Model 2**			**Model 3**		
**N**	**n=48,702**			**n=48,545**			**n=48,545**			**n=48,545**		
	**Events**	**HR (95% CI)**	**p-value**	**Events**	**HR (95% CI)**	**p-value**	**Events**	**HR (95% CI)**	**p-value**	**Events**	**HR (95% CI)**	**p-value**
**Exposure**												
A1c < 7% (ref.)	78	1 (ref.)		78	1 (ref.)	-	78	1 (ref.)		78	1 (ref.)	-
A1c 7-8%	36	0.62 (0.39, 0.99)	0.044	36	0.67 (0.42, 1.07)	0.096	36	0.68 (0.43, 1.08)	0.102	36	0.66 (0.41, 1.05)	0.080
A1c >8%	42	0.68 (0.37, 1.25)	0.219	42	0.88 (0.49, 1.61)	0.690	42	0.89 (0.49, 1.63)	0.708	42	0.82 (0.44, 1.52)	0.534
**Demographic variables**												
Age (continuous)					1.11 (1.09, 1.13)	<0.001		1.11 (1.09, 1.13)	<0.001		1.11 (1.09, 1.13)	<0.001
Non-married (ref.)					1 (ref.)	-		1 (ref.)			1 (ref.)	-
Married					0.83 (0.60, 1.14)	0.238		0.82 (0.60, 1.13)	0.234		0.81 (0.59, 1.12)	0.203
Urban (ref)					1 (ref.)	-		1 (ref.)			1 (ref.)	-
Rural					1.36 (0.92, 2.02)	0.125		1.37 (0.92, 2.03)	0.118		1.37 (0.93, 2.04)	0.114
Service-connected disability <50% (ref.)					1 (ref.)	-		1 (ref.)			1 (ref.)	-
Service-connected disability >=50%					0.50 (0.26, 0.95)	0.035		0.49 (0.26, 0.93)	0.030		0.49 (0.26, 0.94)	0.033
**Clinical variables**												
Annual primary care visit (continuous)								0.98 (0.93, 1.03)	0.394		0.98 (0.93, 1.03)	0.455
Elixhauser comorbidity (continuous)								1.06 (0.96, 1.17)	0.224		1.05 (0.95, 1.15)	0.358

Obesity (BMI ≥30 kg/m^2)								1.17 (0.82, 1.66)	0.386		1.18 (0.83, 1.67)	0.360
**Treatment variables**												
No statin use (ref.)											1 (ref.)	
Statin use											0.61 (0.40, 0.93)	0.021
**T2DM Treatment**												
No medication (ref.)											1 (ref.)	-
Oral medication use only											0.78 (0.44, 1.38)	0.398
Insulin use only											1.46 (0.74, 2.87)	0.275
Both insulin and oral medication use											1.03 (056, 1.92)	0.914

Model 1 = Model 0 + demographic variables (age, race/ethnicity. marital status, location of residence, service-connected disability).

Model 2=Model 1 + clinical variables (Annual primary care visit + Elixhauser comorbidity + Obesity).

Model 3=Model 2 + treatment variable (statin use) + T2DM treatment
