# Supplementary material for: Glycemic Control and Prostate Cancer Mortality Risk in Veterans with Type 2 Diabetes Mellitus
Source: Cancer Res Commun. 2025 Aug 1;5(8):1256–65. doi: 10.1158/2767-9764.CRC-25-0037 (PMC12314478; doi:10.1158/2767-9764.CRC-25-0037)
Supplement: Supplementary Table S3d — Cause-specific competing risk models for the association between time-updated glycemic control and prostate cancer mortality in male veterans with type-2 diabetes among Other [file crc-25-0037_supplementary_table_s3d_suppst3d.pdf]

Supplementary Table S3d: Cause-specific competing risk models for the association between time-updated glycemic control and prostate cancer mortality in male veterans with type-2 diabetes among Other **(with TVC)**

	**Hazard Ratios (HR) and 95% Confidence Intervals (CIs) using Flexible Parametric Models-stpm2 in Stata**											
**Variables**	**Model 0**			**Model 1**			**Model 2**			**Model 3**		
**N**	**n=20,883**			**n=19,705**			**n=19,705**			**n=19,705**		
	**Events**	**HR (95% CI)**	**p-value**	**Events**	**HR (95% CI)**	**p-value**	**Events**	**HR (95% CI)**	**p-value**	**Events**	**HR (95% CI)**	**p-value**
**Exposure**												
A1c < 7% (ref.)	26	1 (ref.)		26	1 (ref.)	-	26	1 (ref.)		26	1 (ref.)	-
A1c 7-8%	18	0.69 (0.31, 1.55)	0.371	18	0.78 (0.35, 1.73)	0.539	18	0.78 (0.35, 1.74)	0.542	18	0.86 (0.39, 1.94)	0.722
A1c >8%	11	0.30 (0.08, 1.12)	0.074	11	0.43 (0.12, 1.60)	0.211	11	0.43 (0.12, 1.60)	0.211	11	0.56 (0.15, 2.09)	0.388
**Demographic variables**												
Age (continuous)					1.12 (1.09, 1.15)	<0.001		1.12 (1.09, 1.15)	<0.001		1.11 (1.08, 1.14)	<0.001
Non-married (ref.)					1 (ref.)	-		1 (ref.)			1 (ref.)	-
Married					1.16 (0.65, 2.08)	0.617		1.18 (0.65, 2.11)	0.590		1.18 (0.65, 2.11)	0.589
Urban (ref)					1 (ref.)	-		1 (ref.)			1 (ref.)	-
Rural					1.65 (0.96, 2.83)	0.068		1.64 (0.95, 2.81)	0.073		1.65 (0.96, 2.83)	0.070
Service-connected disability <50% (ref.)					1 (ref.)	-		1 (ref.)			1 (ref.)	-
Service-connected disability >=50%					1.27 (0.66, 2.47)	0.476		1.24 (0.63, 2.42)	0.537		1.23 (0.63, 2.41)	0.549
**Clinical variables**												
Annual primary care visit (continuous)								1.00 (0.92, 1.08)	0.942		1.00 (0.93, 1.08)	0.972

Elixhauser comorbidity (continuous)								1.06 (0.89, 1.25)	0.523		1.07 (0.90, 1.28)	0.423
Obesity (BMI ≥30 kg/m^2)								0.96 (0.52, 1.78)	0.905		0.99 (0.54, 1.83)	0.983
**Treatment variables**												
No statin use (ref.)											1 (ref.)	
Statin use											0.78 (0.36, 1.67)	0.516
**T2DM Treatment**												
No medication (ref.)											1 (ref.)	-
Oral medication use only											2.60 (0.75, 9.00)	0.132
Insulin use only											2.38 (0.56, 10.03)	0.239
Both insulin and oral medication use											1.04 (0.26, 4.23)	0.951

Model 0 = Unadjusted model
Model 1 = Model 0 + demographic variables (age, race/ethnicity. marital status, location of residence, service-connected disability).
Model 2 = Model 1 + clinical variables (Annual primary care visit + Elixhauser comorbidity + Obesity).
Model 3 = Model 2 + treatment variable (statin use) + T2DM treatment.
