# Supplementary material for: Glycemic Control and Prostate Cancer Mortality Risk in Veterans with Type 2 Diabetes Mellitus
Source: Cancer Res Commun. 2025 Aug 1;5(8):1256–65. doi: 10.1158/2767-9764.CRC-25-0037 (PMC12314478; doi:10.1158/2767-9764.CRC-25-0037)
Supplement: Supplementary Table S4 — Cause-specific competing risk models for the association between time-updated glycemic control and prostate cancer mortality in male Veterans with type-2 diabetes and a stable hemoglobin A1c category throughout follow-up. [file crc-25-0037_supplementary_table_s4_suppst4.pdf]

Supplementary Table S4. Cause-specific competing risk models for the association between time-updated glycemic control and prostate cancer mortality in male Veterans with type-2 diabetes and a stable hemoglobin A1c category throughout follow-up.

	**Hazard Ratios (HR) and 95% Confidence Intervals (CIs) using Flexible Parametric Models-stpm2 in Stata**											
**Variables**	**Model 0**			**Model 1**			**Model 2**			**Model 3**		
**N**	**n=228,212**			**n=226,161**			**N=226,161**			**n=226,161**		
	**Events**	**HR (95% CI)**	**p-value**	**Events**	**HR (95% CI)**	**p-value**	**Events**	**HR (95% CI)**	**p-value**	**Events**	**HR (95% CI)**	**p-value**
**Exposure**												
A1c < 7% (ref.)	666	1 (ref.)		665	1 (ref.)	-	665	1 (ref.)		665	1 (ref.)	-
A1c 7-8%	160	1.88 (1.55, 2.28)	<0.001	159	1.51 (1.24, 1.83)	<0.001	159	1.51 (1.25, 1.84)	<0.001	159	1.45 (1.19, 1.76)	<0.001
A1c >8%	179	0.77 (0.61, 0.99)	0.037	178	1.24 (0.98, 1.57)	0.074	178	1.24 (0.98, 1.57)	0.073	178	1.19 (0.93, 1.53)	0.174
**Demographic variables**												
Non-Hispanic White (ref.)					1 (ref.)	-		1 (ref.)	-		1 (ref.)	-
Non-Hispanic Black					2.17 (1.84, 2.55)	<0.001		2.17 (1.84, 2.56)	<0.001		2.16 (1.83, 2.55)	<0.001
Hispanic					1.20 (0.91, 1.58)	0.194		1.21 (0.92, 1.60)	0.174		1.21 (0.92, 1.59)	0.174
Other					1.12 (0.74, 1.70)	0.594		1.13 (0.75, 1.71)	0.563		1.13 (0.75, 1.72)	0.557
Age (continuous)					1.12 (1.11, 1.12)	<0.001		1.12 (1.11, 1.12)	<0.001		1.12 (1.11, 1.13)	<0.001
Non-married (ref.)					1 (ref.)	-		1 (ref.)			1 (ref.)	-
Married					0.92 (0.81, 1.05)	0.201		0.92 (0.81, 1.05)	0.207		0.92 (0.81, 1.04)	0.182
Urban (ref)					1 (ref.)	-		1 (ref.)			1 (ref.)	-
Rural					1.16 (1.01, 1.32)	0.030		1.16 (1.02, 1.32)	0.029		1.16 (1.02, 1.32)	0.028
Service-connected disability <50% (ref.)					1 (ref.)	-		1 (ref.)			1 (ref.)	-
Service-connected disability >=50%					0.95 (0.77, 1.18)	0.655		0.95 (0.76, 1.18)	0.633		0.95 (0.76, 1.18)	0.001

**Clinical variables**												
Annual primary care visit (continuous)								1.00 (0.98, 1.02)	0.909		1.00 (0.98, 1.02)	0.90
Elixhauser comorbidity (continuous)								1.00 (0.97, 1.04)	0.865		1.01 (0.97, 1.04)	0.790
Obesity (BMI ≥ 30 kg/m^2)								1.12 (0.98, 1.29)	0.101		1.12 (0.98, 1.29)	0.100
**Treatment variables**												
No statin use (ref.)											1 (ref.)	
Statin use											0.79 (0.68, 0.92)	0.003
**T2DM Treatment**												
No medication (ref.)											1 (ref.)	-
Oral medication use only											1.02 (0.86, 1.22)	0.787
Insulin use only											1.24 (0.98, 1.58)	0.076
Both insulin and oral medication use											1.06 (0.83, 1.35)	0.653

Model 0 = Unadjusted model
Model 1 = Model 0 + demographic variables (age, race/ethnicity. marital status, location of residence, service-connected disability).
Model 2 = Model 1 + clinical variables (annual primary care visit + Elixhauser comorbidity + Obesity).
Model 3 = Model 2 + treatment variable (statin use) + T2DM treatment.
