# Supplementary material for: Glycemic Control and Prostate Cancer Mortality Risk in Veterans with Type 2 Diabetes Mellitus
Source: Cancer Res Commun. 2025 Aug 1;5(8):1256–65. doi: 10.1158/2767-9764.CRC-25-0037 (PMC12314478; doi:10.1158/2767-9764.CRC-25-0037)
Supplement: Supplementary Table S5 — Estimated incidence rates (per 1,000) for the association between time-updated glycemic control and prostate cancer mortality for the entire cohort and stratified by race/ethnicity group. [file crc-25-0037_supplementary_table_s5_suppst5.pdf]

Supplementary Table S5. Estimated incidence rates (per 1000) for the association between time-updated glycemic control and prostate cancer mortality for the entire cohort and stratified by race/ethnicity group.

	**Total**			**NHW**			**NHB**			**Hispanic**			**Other**		
**Exposure**	Events	PT	**IR (95% CI)**	Events	PT	**IR (95% CI)**	Events	PT	**IR (95% CI)**	Events	PT	**IR (95% CI)**	Events	PT	**IR (95% CI)**
A1c < 7% (ref.)	1,346	2,512,453	0.54 (0.51, 0.56)	957	1885965	0.51 (0.48, 0.54)	283	410,741	0.69 (0.61, 0.77)	78	139,773	0.56 (0.45, 0.70)	26	68,837	0.38 (0.26, 0.56)
A1c 7-8%	730	1,850,665	0.39 (0.37, 0.42)	570	1400027	0.41 (0.38, 0.44)	104	270,749	0.38 (0.32, 0.47)	36	122,315	0.29 (0.21, 0.41)	18	52,264	0.34 (0.22, 0.55)
A1c >8%	647	1,775,256	0.36 (0.34, 0.39)	448	1212181	0.37 (0.34, 0.41)	143	347,309	0.41 (0.35, 0.49)	42	152,052	0.28 (0.20, 0.37)	11	55,330	0.20 (0.11, 0.36)

IR=incidence rate. NHW=Non-Hispanic White. NHB=Non-Hispanic Black. PT=Person-time.
